# Invariant Natural Killer T Cells As Suppressors of Graft-versus-Host Disease in Allogeneic Hematopoietic Stem Cell Transplantation

**DOI:** 10.3389/fimmu.2017.00900

**Published:** 2017-07-31

**Authors:** Melissa Mavers, Kristina Maas-Bauer, Robert S. Negrin

**Affiliations:** ^1^Divisions of Hematology/Oncology and Stem Cell Transplantation and Regenerative Medicine, Department of Pediatrics, Stanford University, Stanford, CA, United States; ^2^Division of Blood and Marrow Transplantation, Department of Medicine, Stanford University, Stanford, CA, United States

**Keywords:** invariant natural killer T cells, graft-versus-host disease, allogeneic hematopoietic stem cell transplantation, graft-versus-tumor effect, regulatory T cells

## Abstract

Invariant natural killer T (iNKT) cells serve as a bridge between innate and adaptive immunity and have been shown to play an important role in immune regulation, defense against pathogens, and cancer immunity. Recent data also suggest that this compartment of the immune system plays a significant role in reducing graft-versus-host disease (GVHD) in the setting of allogeneic hematopoietic stem cell transplantation. Murine studies have shown that boosting iNKT numbers through certain conditioning regimens or adoptive transfer leads to suppression of acute or chronic GVHD. Preclinical work reveals that iNKT cells exert their suppressive function by expanding regulatory T cells *in vivo*, though the exact mechanism by which this occurs has yet to be fully elucidated. Human studies have demonstrated that a higher number of iNKT cells in the graft or in the peripheral blood of the recipient post-transplantation are associated with a reduction in GVHD risk, importantly without a loss of graft-versus-tumor effect. In two separate analyses of many immune cell subsets in allogeneic grafts, iNKT cell dose was the *only* parameter associated with a significant improvement in GVHD or in GVHD-free progression-free survival. Failure to reconstitute iNKT cells following allogeneic transplantation has also been associated with an increased risk of relapse. These data demonstrate that iNKT cells hold promise for future clinical application in the prevention of GVHD in allogeneic stem cell transplantation and warrant further study of the immunoregulatory functions of iNKT cells in this setting.

## Introduction

Hematopoietic cell transplantation (HCT) remains the only curative treatment option for patients with many hematologic malignancies and hematologic disorders. Despite many improvements to support patients following transplantation, graft-versus-host disease (GVHD) continues to be one of the leading causes of morbidity and mortality (1). GVHD is characterized by a dysregulation of donor immune cells leading to a massive alloreactive T cell proliferation and destruction of host tissues (2). To better understand the mechanisms underlying this dysregulation and potentially improve the outcome following HCT, different immune regulatory cell populations such as regulatory T cells (Tregs) and invariant natural killer T (iNKT) cells have been extensively studied. This review will focus on the immune biology of iNKT cells in the transplant setting.

Invariant natural killer T cells are a rare subset of T lymphocytes which are characterized by the coexpression of natural killer and T cell markers. They express a T cell receptor (TCR) which is semi-invariant (Vα14Jα18 pairing with a limited selection of beta chains in mice and Vα24Jα18 typically pairing with Vβ11 in humans) and which recognizes glycolipid antigens presented by the non-polymorphic MHC Class I-like molecule CD1d with high affinity (3).

**Figure 1:**
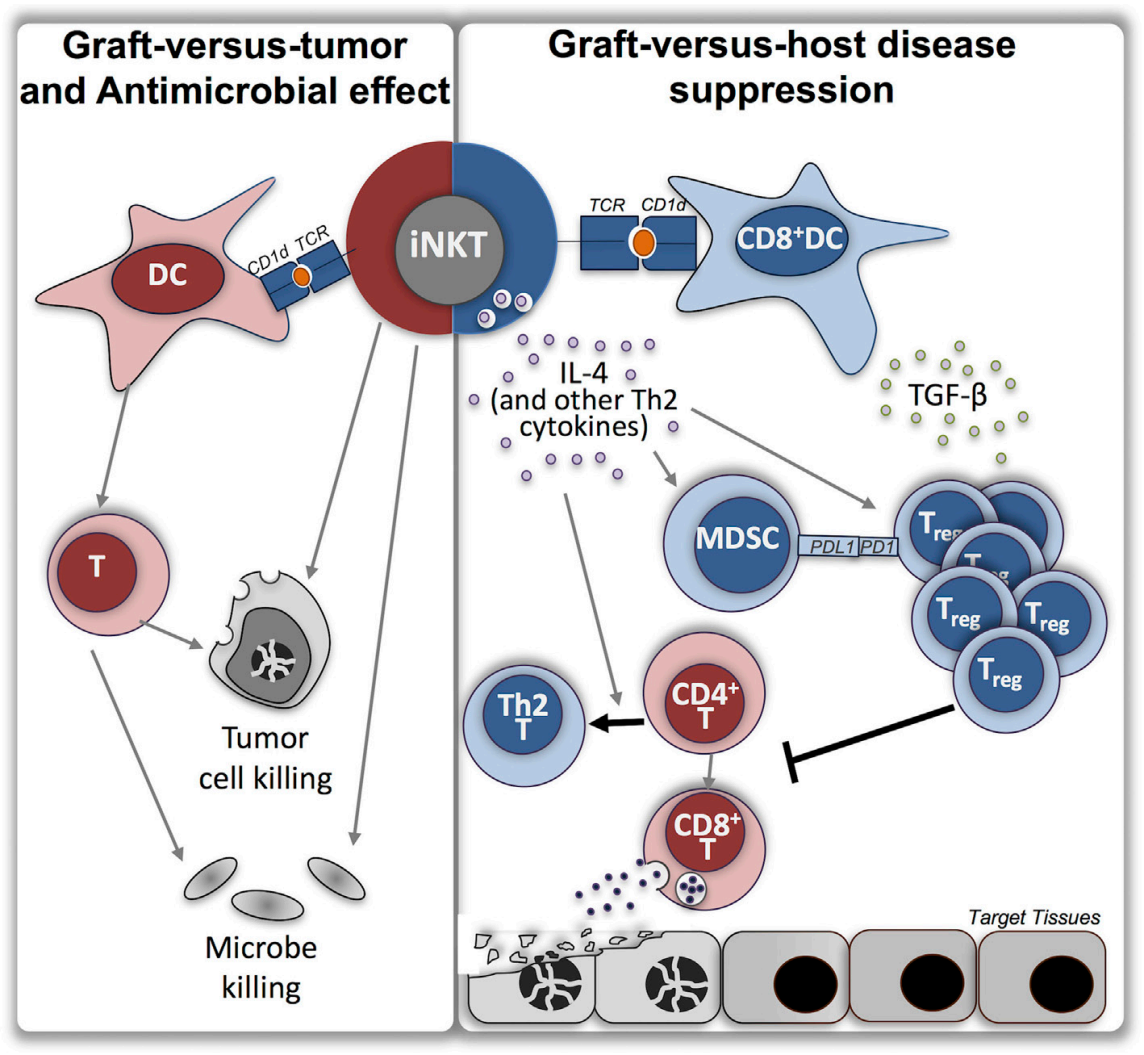
Roles of invariant natural killer T cells in allogeneic stem cell transplantation. Depicted are the variety of roles for iNKT in allogeneic stem cell transplantation which have been demonstrated in multiple studies, including graft-versus-tumor effect, antimicrobial effect, and suppression of graft-versus-host disease (GVHD). Purported mechanisms by which iNKT may function to suppress GVHD are shown. Red color represents inflammatory phenotype, blue color represents suppressive or anti-inflammatory phenotype, orange circle represents endogenous lipid antigen, foreign lipid antigen, or exogenous synthetic lipid antigen [i.e., alpha-galactosylceramide (α-GalCer)]. iNKT, invariant natural killer T cells; TCR, T cell receptor; DC, dendritic cell; IL, interleukin; TGF, transforming growth factor; MDSC, myeloid-derived suppressor cell; Treg, regulatory T cell; T, T lymphocyte.

Despite their rarity, iNKT cells exert potent immunomodulatory functions bridging the innate and adaptive immune systems by rapidly producing vast amounts of cytokines and chemokines. This results either in enhanced immune responses (i.e., defense against pathogens, immunosurveillance in cancer) via the production of Th1 cytokines such as interferon (IFN)-γ or in suppression of autoimmune and alloimmune reactions by the production of interleukin (IL)-4 and IL-10 (4, 5) (Figure 1).

## Murine Studies

### Host iNKT Cells Protect from GVHD

During the last two decades multiple studies have focused on the role of iNKT cells in the context of allogeneic HCT utilizing different animal models. One approach from the group of Strober et al. revealed that reduced intensity conditioning (RIC) with total lymphoid irradiation (TLI) and antithymocyte globulin (ATG) results in a relative expansion of host iNKT cells which protect from GVHD, while preserving the graft-versus tumor effect (6, 7). The protection from GVHD was associated with a bias in donor T cell polarization to a Th2 cytokine pattern. The same group also demonstrated that host iNKT cells induce a donor Treg expansion and that these Tregs are crucial to protect from GVHD (8). The Treg proliferation was IL-4 dependent as it was lost when IL-4 deficient recipients were used. Another study investigating the effect of residual host NKT cells in a major mismatch transplantation model with total body irradiation (TBI) found that these cells are essential for the protection from GVHD but can also cause a delay in engraftment and, under certain conditions, graft rejection (9).

### Stimulation of iNKT Cells with Alpha-Galactosylceramide (α-GalCer) Protects from GVHD

Treatment of recipient mice with a synthetic iNKT TCR ligand α-GalCer (in aqueous or liposomal form) on the day of transplantation significantly reduced mortality and morbidity from GVHD (10–13). One study showed that this effect was abrogated if NKT cell-deficient CD1d knockout (CD1d^−/−^) or IL-4^−/−^ recipient mice were used, indicating that host iNKT cells and their production of IL-4 play a critical role in tolerance induction (11). Furthermore, in the same study it was shown that tolerance induction also failed if the donors were signal transducer and activator of transcription (STAT)6 deficient. This suggests that the protection from GVHD is dependent on the Th2 polarization of donor T cells mediated by STAT6-dependent mechanisms. Another study confirmed that the activation of iNKT with the liposomal form of α-GalCer and a CD40-CD40 ligand blockade lead to the establishment of a mixed chimerism and provides tolerance in a murine model with combined allogeneic bone marrow transplantation (BMT) and heart transplantation (13). Together with the protection from GVHD and the establishment of a mixed chimerism, an activation of Tregs and high amounts of Th2 cytokines in the recipient mice was observed. In line with this, Duramad et al. observed a dose-dependent expansion of Tregs in spleen, lymph nodes, and bone marrow after administration of α-GalCer on the day of HCT (12). Interestingly the protective effect of α-GalCer was abrogated if the donors were Treg depleted. Further, in a model of chronic GVHD (cGVHD) the administration of α-GalCer is capable of reversing and preventing cGVHD (14). As described below, treatment with α-GalCer for the protection of GVHD is the first approach that has been successfully translated into the clinic and is currently being investigated in clinical trials.

### Adoptive Transfer of iNKT Cells Protects from GVHD

Another approach to harnessing the immunoregulatory abilities of iNKT cells is the adoptive transfer of either recipient-type, donor-type, or third party iNKT cells in the allogeneic transplantation setting. Several studies by our group and others convincingly demonstrated that the adoptive transfer of iNKT cells leads to a decrease in acute GVHD without losing the graft-versus-tumor (GVT) effect (7, 9, 15–17). In these studies, adoptively transferred donor CD4^+^ iNKT expanded in secondary lymphoid organs and migrated to GvHD target organs, similar to conventional T cells (15). Interestingly, we have shown that extremely low iNKT cell numbers (i.e., 5 × 10^4^) and even cells from a third-party source are capable of these effects (15–17). Additionally, the adoptive transfer of donor iNKT cells is able to prevent and even reverse cGVHD in a minor-mismatch BMT model. This effect was also dependent on the production of IL-4 by iNKT cells and the presence of Tregs (14).

In an effort to make iNKT cells more accessible in the clinic, *ex vivo* expansion before adoptive transfer has been explored. Several studies demonstrated that it is feasible to expand iNKT cells *in vitro* with a combination of α-GalCer and IL-2. In these studies, the expanded iNKT cells provide protection from GVHD which is dependent on the production of IL-4 by iNKT cells (18, 19).

### Mechanisms of iNKT Cell Function in Murine HCT

Most of the studies described above had two intriguing findings in common. First, iNKT cells give rise to a bias in donor T cell polarization toward a Th2 cytokine pattern with significantly reduced production of IFN-γ and tumor necrosis factor (TNF)-alpha (6, 13, 15, 17), and some even showed that the proliferation of conventional T cells was diminished (6, 16). Second, the survival benefit of mice treated with TLI/ATG, donor iNKT cells, or α-GalCer was accompanied by an expansion of Tregs (12–14, 16, 17, 20). Tregs have been shown to function as potent immune suppressors in the context of allogeneic transplantation and are capable of both inhibiting GVHD as well as preserving the GVT effect (21–23). There is compelling evidence that the mechanism by which iNKT cells suppress GVHD is through the expansion of Tregs. Accordingly, it was shown in different murine transplantation models that cytokines such as IL-4 produced by iNKT cells play an important role in enhancing Treg function and that depletion of Tregs leads to a loss of function of iNKT cells (14, 17, 20). Interestingly, it was also demonstrated that Tregs are not capable of inducing tolerance in a model of combined marrow and organ transplantation if the recipient is iNKT cell deficient (20). Another hypothesis to support the latter findings is that other cell populations, such as myeloid-derived suppressor cells (MDSCs) or CD8^+^ dendritic cells (DC), play an important role in the interplay between iNKT cells and Tregs.

Myeloid-derived suppressor cells are a heterogenous cell subset known to play a major role in the regulation of immune responses in cancer and other pathological conditions (24), and several studies have shown that they have the potential to inhibit GVHD (25, 26) and to induce Treg proliferation after HCT in PDL1-dependent manner (25, 26). Moreover, we demonstrated that MDSCs can work as a facilitator between iNKT cells and Tregs in a murine allogeneic BMT model with adoptive transfer of donor iNKT cells (17). In this model, certain subsets of MDSCs were shown to expand shortly after transplantation and, if depleted, the protective effect of the transferred donor iNKT cells was lost. Furthermore, in the same model, MDSCs were also crucial to mediate the iNKT cell-induced expansion of Tregs as the depletion of MDSCs led to a depletion of Tregs (17). In another model with combined bone marrow and heart transplantation, MDSCs were crucial to promote tolerance and chimerism and their activation was dependent on host iNKT cells and their production of IL-4 (27).

The second cell population, which has recently come to attention, is CD8α^+^ DCs. It was shown previously that these cells are the major DC subset to present a variety of glycolipids through the CD1d molecule to iNKT cells leading to their activation (28). Furthermore, it is known that CD8α^+^205^+^DCs induce Tregs in a transforming growth factor-beta (TGF-β) and retinoic acid-dependent manner (29) and that they can exert immunosuppressive characteristics in specific situations yet are crucial to promote the GVT effect (29, 30). One group also found that there is an aggravated course of GVHD if this subset of DCs is missing (31). In addition, the total number of Tregs and levels of TGF-β are significantly lower when CD8^+^ DCs are not present. CD8^+^ DCs induced iNKT cells to secret IL-4, IL-13, and IFN-γ with a Th-2 bias (32). In return these tolerogenic iNKT cells altered the differentiation of CD8^+^ DCs and suppressed graft rejection *in vivo* demonstrating elegantly that interactions between tolerogenic CD8^+^ DCs and iNKT cells are required to induce tolerance.

## Human Studies

In addition to these murine experiments, a number of human studies have also shown a role for iNKT in suppressing GVHD. Although largely correlative, these studies demonstrate the power of iNKT cells and the need for further research. In these reports, iNKT were defined as CD3^+^Vα24^+^Vβ11^+^, except where indicated.

### Persistence and Rapid Recovery of iNKT Cells following Allogeneic HCT Are Associated with Reduced GVHD

One of the earliest studies delineating iNKT reconstitution following allogeneic HCT demonstrated a correlation between increased peripheral blood iNKT count and reduced acute and chronic GHVD (33). In this study iNKT were enumerated in the peripheral blood starting on day +30 following transplantation of 106 patients undergoing HCT without T-depletion following myeloablative conditioning (MAC). Donors included a mix of matched unrelated donors (MUDs) and matched related donors (MRD). In a multivariate analysis, stem cell source was the only variable associated with iNKT content in the peripheral blood post-transplantation. Following peripheral blood stem cell transplantation (PBSCT), iNKT cells were reconstituted within 1 month, while after BMT, iNKT were not fully reconstituted within 1 year (and all iNKT were <10% recipient chimerism in four analyzed patients). In BMT, but not PBSCT, the number of total, CD4^+^, and CD4^−^ iNKT were significantly reduced in patients with aGVHD, and total and CD4^+^ iNKT (as well as iNKT/T cell ratio) were significantly reduced in extensive cGVHD. Importantly, steroids had no impact on iNKT counts. Although limited by quite high numbers of missing values, this study represents the first human report to explore the link between iNKT recovery and development of GVHD.

An early human study from our group translated the encouraging murine TLI/ATG results in 37 patients receiving human leukocyte antigen-matched (MRD or MUD) granulocyte colony-stimulating factor-mobilized PBSCT for hematologic malignancies (34). Only two patients developed aGVHD (one was only grade I), considerably lower than previously reported for RIC regimens, and only seven developed extensive cGVHD. Importantly, 62% either converted to or remained in CR. Interestingly, production of IL-4 (but not IFN-γ, IL-2, or TNF-α) was markedly elevated in CD4^+^ T cells from patients who underwent TLI/ATG conditioning as compared to conventional RIC or normal subjects. In the five patients for whom peripheral blood iNKT count was measured following conditioning, iNKT percentage was increased by a factor of 10. Building on this success, a second report of 111 additional patients, including a few mismatched unrelated donors (MMUD) demonstrated that the overall probability of aGVHD was substantially lower than typically observed with RIC at 5.4%, and slightly lower for extensive chronic GVHD at 28% (35). One limitation was that 19% of patients developed mixed chimerism, associated with higher risk of disease relapse or progression. The overall risk of relapse/progression was around 60%, though many were salvaged with donor lymphocyte infusion. Non-relapse mortality (NRM) was 3% suggesting excellent tolerability; indeed, death from infection was extremely low possibly related to less need for immunosuppression given the low GVHD rates. These findings were independently corroborated when TLI/ATG was used in a multicenter study of 45 patients (36) and when compared with TBI/fludarabine in a randomized study (37).

Interestingly, the effect of ATG on iNKT preservation and GVHD reduction does not appear to extend to the myeloablative setting. Immune cell reconstitution was analyzed starting at day +40 in 65 patients undergoing MAC prior to PBSCT with or without ATG (38). In this setting, the inclusion of ATG led to a significantly *decreased* number of iNKT cells (defined as CD3^+^CD56^+^Vα24Jα18^+^Vβ11^+^) at day +40, though by day +100 no difference was noted. The ATG group also had a dramatically lower incidence of aGVHD with no change in cGVHD. Another study failed to find an association of iNKT with GVHD in a MAC plus ATG setting (39). Similarly, a third study of MAC plus ATG conditioning evaluated iNKT after conditioning and starting at day +7 (40). Normalization of iNKT to healthy control levels had not occurred by day +730, though CD4^−^ iNKT were recovering faster than CD4^+^ iNKT. Interestingly, CD4^−^ iNKT cells were significantly *higher* on d28 in patients who ultimately developed GVHD versus those did not.

However, in a study of 71 heterogenous patients (MAC and RIC, T cell-depleted and non-depleted, mix of donor types and cell sources), early post-transplantation iNKT recovery [defined as CD3^+^CD1d tetramer (PBS57)^+^] predicted aGVHD and overall survival (OS) (41). iNKT/T ratio was monitored post-transplant and a threshold of 1 × 10^−3^ appeared to discriminate patients into low and high groups predictive of aGVHD. In multivariate analysis, high iNKT/T ratio was significantly associated with absence of aGVHD (hazard ratio 0.12), and OS was also significantly improved (90 versus 52% at 2 years). Significant reduction in NRM (6.1 versus 36.7%) was noted to be due to fewer deaths from aGVHD and infection. Impressively, the d15 ratio (with a cutoff of 0.58 × 10^−3^) could discriminate the risk of aGVHD with an area under the curve of 0.812 (odds ratio 0.06 for aGVHD in high iNKT/T group). Interestingly, CD4^+^ iNKT chimerism of five patients revealed all to be of donor origin.

### Recovery of iNKT Cells Is Also Associated with Enhanced GVT Effect

One study evaluated iNKT reconstitution (CD3^+^Vα24Jα18^+^) in cord blood and following umbilical cord blood (UCB) transplantation in 33 patients with high-risk acute myeloid leukemia (AML) following RIC (42). While iNKT cells were reduced after conditioning compared to healthy controls, a transient increase in frequency was observed in the first 3 months post-transplant (which was not seen in other T cell populations). In the first few months, most iNKT were CD4^+^, similar to that observed in UCB, which fell to healthy adult control levels by 12 months. CD45RO expression remained high throughout (with low CD45RA), suggesting a primed/memory phenotype similar to UCB, while CD62L and CCR7 fell over time, suggesting progression from a central memory phenotype toward more effector/memory tissue-homing. Early post-transplant iNKT cells were also enriched for CD69 which decreased over time, suggesting early activation. Production of IFN-γ and IL-4 were both substantially reduced and granulocyte–macrophage colony-stimulating factor was substantially increased in UCB and in early post-transplant samples compared to healthy adults, which all normalized by 6 months post-transplant. Although cytotoxicity of iNKT against CD1d-transfected AML cell lines and primary AML blasts was significantly reduced in UCB and early post-transplant, it reached healthy adult control levels within 6 months. This study highlights the reconstitution process of iNKT cells following UCB transplantation and begins to elucidate the mechanisms by which GVT may occur.

Invariant natural killer T cell reconstitution was further studied in a group of pediatric patients receiving T cell depleted haploidentical transplants for hematologic malignancies (43). Twenty-two patients were followed longitudinally from day +30 to 18 months; another 11 underwent cross-sectional analysis at 2–6 years post-transplant. In this setting, iNKT cells emerged in peripheral blood around 3 months post-transplant and reached levels similar to age-matched healthy children by 18 months. The iNKT cell profile initially resembled that of cord blood: predominantly CD4^+^CD161^−^. CD4^−^ iNKT began to emerge about 2–4 months later than CD4^+^ iNKT and CD161^+^ cells emerged over time in both populations. IFN-γ production reached levels close to healthy adults by 6 months in CD4^−^ iNKT, and both populations could produce IL-4 normally. This study also strikingly demonstrated that delayed iNKT reconstitution was significantly associated with relapse. iNKT remained essentially undetectable during the entire 18-month follow-up in the eight patients who experienced relapse, while absolute iNKT cell counts were significantly higher in all patients who maintained remission. Although a decreased absolute number of T cells was also significantly associated with relapse, in terms of frequency only a lower iNKT cell frequency was significantly associated with relapse (19 iNKT/10^6^ T cells in patients who relapsed versus 107 iNKT cells/10^6^ T cells in those who did not) (44). The authors suggest that monitoring of iNKT cell reconstitution post-transplant and adoptively transferring donor iNKT cells in those patients failing to reconstitute may be a method by which relapse could be prevented.

### Increased Number of iNKT Cells in the Allogeneic Graft Product Is Associated with Reduced Acute GVHD

In addition to the above studies demonstrating that persistence and/or rapid recovery of iNKT cells following transplantation correlate with reduced GVHD, a number of studies have evaluated the graft content of various cell populations and potential impact on clinically meaningful outcomes. The frequency of lymphocyte subsets in cryopreserved samples of 78 sibling donor non-T-depleted PBSCT grafts for patients undergoing mostly MAC were analyzed (45). In multivariate regression analysis, CD4^−^ iNKT cell dose and chronic myeloid leukemia diagnosis were the only factors associated with aGVHD, with a RR of 4.27 for CD4^−^ iNKT dose below the median (0.031 × 10^6^/kg). Interestingly, graft Treg dose did not predict for grades II–IV aGVHD. The impact of iNKT cell dose on chronic GVHD, relapse, or survival was not assessed. Further, CD4^−^ iNKT cells consistently suppressed proliferation in a mixed lymphocyte reaction with autologous T cells and allogeneic stimulator cells in a dose-dependent manner, and suppressed IFN-γ secretion.

Another study characterized over 25 immune cell subsets within cryopreserved grafts of 80 patients undergoing PBSCT from a mix of MRD, MUD, and MMUDs (46). Most patients received RIC and the vast majority received *in vivo* T cell depletion with ATG. The dose of iNKT cells (CD3^+^Vα24^+^) was the only factor with a significant impact on GVHD-free progression-free survival (GPFS) in multivariate analysis. The 2-year GPFS was significantly increased in patients receiving greater than the median number of donor iNKT cells (0.11 × 10^6^/kg) at 49% compared to 22% in patients receiving fewer than the median number, although OS was not significantly different. The findings were primarily due to an increased incidence of relapse in the patients receiving fewer than the median iNKT cell dose, as there was no significant difference in NRM, grades III–IV acute GVHD, or severe chronic GVHD. As noted in the manuscript, the use of ATG leading to very low rates of aGVHD overall (20–25%) may explain the lack of association of increased iNKT with reduction in acute GVHD.

A third study investigated 15 immune populations from a mix of fresh and cryopreserved graft products for 117 transplants from a variety of donors (MRD/MUD/MMUD), sources (PBSCT/BMT), and conditioning regimens (MAC/RIC, with about half receiving T-depletion with ATG) (47). Doses of iNKT [CD3^+^CD1d-tetramer(PBS57)^+^] were noted to be higher in PBSC than BM grafts owing to the one log higher total T cell content. Although lower total and CD4^−^ iNKT frequency was significantly associated with the occurrence of aGVHD in BMT and PBSCT, lower CD4^−^ iNKT expansion factor was only associated with aGVHD in the PBSCT group. Importantly, this remained true when the expansion of iNKT cells was analyzed in donor peripheral blood *prior* to mobilization and at defined timepoints in patients post-transplant. The expansion factor was not predictive of relapse rate, cGVHD, NRM, or OS. Although, as the authors recognize, the ability to implement the expansion technique as a GVHD predictive tool across all centers is unlikely, this work highlights the critical need for high frequencies of functional iNKT cells for suppression of aGVHD. It also raises the question of whether iNKT cell dose or frequency relative to total T cells (or, indeed, expansion capacity) is the most important value in predicting aGVHD, which remains to be fully answered.

Interestingly, another study of immune cell content in 238 allografts for a very heterogenous patient population showed a significant association of increased iNKT content with *increased* risk of cGVHD in a univariate analysis (48). However, a non-standardized definition of iNKT cells was used (CD3^+^CD56^+^CD16^−^CD117^−^) and this finding did not pan out in multivariate analysis. The same study also found that increased iNKT cell content was associated with lower risk of relapse in AML/MDS patients.

### Therapeutic Expansion of iNKT Compartment Is Well Tolerated with Lower GVHD Rates in Responders

Because of this strong evidence for GVHD suppression by iNKT cells, attempts to intentionally expand the iNKT cell compartment in allogeneic HCT patients have begun. A recently published phase 2A study explored pharmacologic expansion using a single dose of RGI-2001, the liposomal formulation of α-GalCer, on the day of transplantation with two dosing cohorts (identified from phase 1 testing) for a total of 29 patients (most undergoing RIC with PBSC grafts) (49). A total of 11 serious adverse events (two grade IV) were reported, regardless of causality. Immune cells were monitored prior to transplant and weekly for the first month with no significant changes in iNKT noted (remained <3% of total lymphocytes). However, similar to the iNKT-mediated Treg expansion noted in mice (16), four patients from each cohort had significant expansion of absolute number and frequency of Tregs (CD4^+^CD25^hi^CD127^low^). These responders were enriched for patients who had received sirolimus with their GVHD prophylaxis (though not statistically significant), suggesting a synergistic effect (although an isolated effect of sirolimus cannot be entirely ruled out). Importantly, rates of grades II–IV aGVHD were 12.5% in responders and 52.4% in non-responders, though as noted by the authors based on the study design conclusions cannot be drawn as to whether RGI-2001 reduces GVHD incidence. Future work optimizing the dosing strategy, comparing patients receiving RGI-2001 to those who do not, and investigating why some respond and others do not will be required. Despite these limitations, this study represents a critical first step in the intentional boosting of iNKT cells to suppress GVHD.

## Conclusion and Future Directions

As demonstrated in the aforementioned murine and human studies, iNKT cells may be the holy grail sought in stem cell transplantation that is capable both of suppressing allogeneic immune reactions (GVHD) and enhancing anti-tumor immune reactions (GVT). The murine studies all demonstrated a strong benefit of iNKT cells on GVHD reduction. Although a minority of human studies (primarily in the MAC plus ATG setting) did not find a significant association between iNKT and GVHD, or even found higher iNKT associated with worse GVHD (38–40), most found that increased iNKT cell content of the graft or of peripheral blood post-transplantation was associated with GVHD suppression. One should keep in mind, however, that these are correlative studies. Indeed, cause-and-effect cannot be determined for those studies evaluating iNKT content of peripheral blood, and the possibility of evolving GVHD causing lowered iNKT numbers in peripheral blood (perhaps due to increased target tissue homing) cannot be excluded. However, the studies revealing an association between higher iNKT counts (in the graft or peripheral blood) and improved NRM and survival (41, 46) suggest that, in general, iNKT cells exert an overall protective effect. The extensive murine data support this as well. Indeed, given the broad functions of iNKT cells, increased numbers may be beneficial not just for GVHD suppression, but also infection control and reduction in relapse (Figure 1).

Despite these promising results, however, a number of questions remain unanswered. First is whether donor iNKT, recipient iNKT, or both are critical for these functions. Although the results from TLI/ATG conditioning studies suggest a role for host iNKT in suppression of GVHD, the importance of donor graft iNKT cell content in other studies, as well as iNKT chimerism revealing donor origin, implicate the role of donor iNKT cells. One could envision that donor iNKT cells are most critical in a MAC setting, while both donor and recipient iNKT play a role in RIC, but more study is needed to tease apart their individual contributions. Second, is whether certain iNKT cell subsets are more functional in the transplant setting and/or whether different subsets serve different (and yet equally critical) functions in transplant patients. The results of studies included in this review are not always congruous in this regard. In the murine adoptive transfer studies, our group found that CD4^+^ iNKT suppressed aGVHD in a major mismatch model (16, 17). However, two human studies have found in multivariate analyses that CD4^−^, not CD4^+^, iNKT graft content was significantly associated with reduced aGVHD (45, 47). Conversely, an increased number of CD4^+^ iNKT in post-transplant peripheral blood was significantly associated with reduced cGVHD (increased CD4^−^ iNKT were also associated with reduced cGVHD, but did not reach significance) (33). The discrepancy between mouse and human studies (and indeed, among human data) as to whether the CD4^+^ or CD4^−^ iNKT population is best suited to reduce GVHD (and possible differences between acute and chronic GVHD) highlights the need for further study. Recently, several subsets of iNKT cells have been described skewing toward phenotypes resembling Th1 cells, Th2 cells, and Th17 cells [reviewed in Ref. (5)], and even Treg-like iNKT which constitutively produce IL-10 (50). CD4^−^CD8^+^ iNKT have also been described in humans, but not mice, and exhibit potent cytotoxic functions (51). The contribution of each of these subsets in the setting of stem cell transplantation remains to be determined, and a better understanding of their physiologic and pathologic functions, as well as their degree of plasticity, will inform future work. In addition, the ability of CD4^+^ iNKT cells to produce some Th1-type cytokines and vice versa (51) calls into question whether distinguishing subsets of iNKT based on expression of CD4 alone and thereby assigning differing functions is appropriate. A third question is how to boost iNKT numbers in patients receiving grafts containing low iNKT numbers or with poor post-transplantation iNKT reconstitution, and indeed these cutoffs remain to be clearly established (and may differ depending on transplant conditions). However, these studies begin to identify an iNKT cell dose in the graft, or in peripheral blood as the population is reconstituting, that is associated with lower GVHD risk. This will aid clinical trial design for the adoptive transfer of iNKT cells. Several approaches have been investigated in mice to intentionally use iNKT cells to dampen GVHD while preserving the GVT effect. For instance, iNKT cells were expanded and their immunoregulatory abilities enhanced by treating recipient mice with α -GalCer on the day of transplantation (10–12). In addition, iNKT cells from different origins (host, donor, third party) were each shown to be effective in reducing acute and chronic GVHD when adoptively transferred together with the graft (7, 9, 14–16). Furthermore, iNKT cells can be expanded *ex vivo* with IL-2 and α-GalCer and adoptively transferred for GVHD suppression (18, 19). Most of these studies also demonstrated a conservation of the GVT effect. There is convincing evidence in mice that iNKT cells work through the expansion of Tregs, and the underlying biological mechanism is currently under investigation. IL-4 production by iNKT cells or facilitators such as MDSCs or CD8^+^ DCs are possibilities. The findings that third party iNKT cells are as functional as donor-derived cells for the suppression of GVHD in mice and that the ability to expand these cells has been demonstrated sets the stage for clinical translation.

The first approach being investigated in humans utilizes delivery of α-GalCer to recipients at the time of transplantation (49). Though the choice of most appropriate transplant setting and optimal dosing strategy remains to be determined, phases 1 and 2A trials have revealed this approach to be safe. Given the results with other approaches in mice, it would be exciting to pursue these additional strategies in humans. The non-polymorphic nature of the CD1d molecule, as well as the ability of iNKT cells to be activated in non-TCR-dependent ways, make adoptive transfer of iNKT cells a feasible approach. Correlative biologic evaluations must accompany all clinical trial designs to determine the effect that any approach has not only on iNKT cell number, but also functional skewing.

Despite these lingering questions, harnessing the power of iNKT cells remains an attractive approach to improving HCT outcomes. Although extremely rare in number, iNKT cells may represent the most versatile and critical cell population for suppressing GVHD, fighting infection, and reducing relapse in patients undergoing allogeneic hematopoietic cell transplantation.

## Author Contributions

MM and KB contributed equally to this manuscript. MM and KB did the literature study, wrote the manuscript, and critically revised the manuscript. RN provided senior supervision, helped in writing, and critically revised the manuscript. All coauthors approved the final manuscript.

## Conflict of Interest Statement

The authors declare that the research was conducted in the absence of any commercial or financial relationships that could be construed as a potential conflict of interest.

## Abbreviations

α-GalCer, alpha-galactosylceramide; aGVHD, acute graft-versus-host disease; AML, acute myeloid leukemia; ATG, antithymocyte globulin; BMT, bone marrow transplantation; cGVHD, chronic graft-versus-host disease; DCs, dendritic cells; GCSF, granulocyte colony stimulating factor; GPFS, GVHD-free progression-free survival; GVHD, graft-versus-host disease; GVT, graft-versus-tumor; HCT, hematopoietic cell transplantation; IFN, interferon; IL, interleukin; iNKT, invariant natural killer T; MAC, myeloablative conditioning; MDSCs, myeloid-derived suppressor cells; MMRD, mismatched unrelated donor; MRD, matched related donor; MUD, matched unrelated donor; NRM, non-relapse mortality; OS, overall survival; PBSCT, peripheral blood stem cell transplantation; RIC, reduced intensity conditioning; STAT, signal transducer and activator of transcription; TBI, total body irradiation; TCR, T cell receptor; TGF, transforming growth factor; TLI, total lymphocyte irradiation; TNF, tumor necrosis factor; Tregs, regulatory T cells; UCB, umbilical cord blood.
